# War Trauma and Cancers

**Published:** 1918-12

**Authors:** 


**War-Trauma and Cancers.** By L. Bérard. *Bulletin de l'Académie de Médecine*, July 16, 1918.

Trauma seems to interfere with the development of cancers, according to some definite rules.

1) Epithelial tumors sometimes appear after repeated irritating causes acting upon tissues previously injured; upon scars, for instance, or parts inflamed by radio-dermitis, or burns, etc.

2) Certains tumors, especially those of a conjunctivo-vascular variety, may develop after one single strong trauma.

Among 71 cases of cancers, observed by the author among soldiers, 7 seem to be in intimate connection with some trauma. For instance, an epithelioma of the scalp was seen to develop on an ancient scar, just where this was irritated by the helmet. A fibrosarcoma appeared under the pressure of some shoulder-straps in a part of the shoulder previously injured. Three cancers of the sarcoma variety followed horse-kicks, and seemed really to be caused directly by those accidents.

